# Circulating exosomal biomarkers in schizophrenia: Diagnostic utility and clinical implications

**DOI:** 10.1192/j.eurpsy.2026.12165

**Published:** 2026-07-31

**Authors:** N. Igor, B. Larisa

**Affiliations:** Mental Health, Medical Psychology and Psychotherapy, USMF, Chișinau, Moldova, Republic of

## Abstract

**Introduction:**

Validated blood biomarkers for schizophrenia remain unavailable. Circulating exosomes (extracellular vesicles, EVs) carrying microRNAs and proteins provide measurable signals. In first-episode psychosis, microRNA panels show high diagnostic accuracy (sensitivity 76–96%, specificity 78–96%, AUC up to 0.94). Brain-derived exosomes reveal disrupted signalling and mitochondrial dysfunction correlating with symptoms. Clinical studies suggest exosomes are promising biomarkers for early diagnosis, therapy monitoring, and pathogenetic research.

**Objectives:**

To summarize diagnostic characteristics (sensitivity [Se], specificity [Sp], AUC) of exosomal biomarkers in schizophrenia, quantitatively assess their performance, and examine Se–Sp relationships.

**Methods:**

Due to scarce randomized clinical trials (RCTs) and limited Se/Sp data, all peer-reviewed studies (2019–2024) were included regardless of design (case–control, cross-sectional, cohort, including three RCTs). Criteria: (1) schizophrenia patients with healthy or clinical comparators; (2) reported Se/Sp and/or AUC. Descriptive statistics (medians/ranges), linear regression (Sp on Se), and Pearson correlation (95% CI) were performed. Between-test differences were assessed with ANOVA, Levene’s test, and Shapiro–Wilk. No individual data were pooled.

**Results:**

Across 12 studies, median Se was 85.35% (range 64.9–100), Sp 86.50% (60.2–100), and AUC 90.25% (66.1–100). Se correlated positively with Sp (r = 0.76, 95% CI 0.34–0.93, p < 0.01); regression slope β = 0.81 (p = 0.004), R² = 0.59. Between-test differences were nonsignificant (ANOVA F = 0.63, p = 0.538). An 11-miRNA exosomal signature achieved AUC 0.94 (95% CI 0.88–1.00) in training and 0.753 (0.61–0.90) in testing. Complement-enriched exosomal proteins (C3, C4, C4BPA, PROS1) discriminated schizophrenia from controls (AUC 0.895) and from bipolar disorder (0.966). Multicenter data support generalizability.

**Conclusions:**

Exosomal biomarkers demonstrate high diagnostic performance (AUC ≈ 0.90; Se/Sp ≈ 85–87%) with consistent Se–Sp association. Panels combining miRNAs and complement proteins are especially promising. Limitations include design heterogeneity and few RCTs. Harmonized EV protocols, blinded multicenter validation, and studies of treatment-response prediction are needed.

**Disclosure of Interest:**

None Declared

